# Glioblastoma Microenvironment: From an Inviolable Defense to a Therapeutic Chance

**DOI:** 10.3389/fonc.2022.852950

**Published:** 2022-03-02

**Authors:** Vincenzo Di Nunno, Enrico Franceschi, Alicia Tosoni, Lidia Gatto, Stefania Bartolini, Alba Ariela Brandes

**Affiliations:** ^1^ Department of Oncology, AUSL Bologna, Bologna, Italy; ^2^ Nervous System Medical Oncology Department, IRCCS Istituto delle Scienze Neurologiche di Bologna, Bologna, Italy

**Keywords:** microenvironment, glioblastoma, macrophages, neurons, immune-system

## Abstract

Glioblastoma is an aggressive tumor and is associated with a dismal prognosis. The availability of few active treatments as well as the inexorable recurrence after surgery are important hallmarks of the disease. The biological behavior of glioblastoma tumor cells reveals a very complex pattern of genomic alterations and is partially responsible for the clinical aggressiveness of this tumor. It has been observed that glioblastoma cells can recruit, manipulate and use other cells including neurons, glial cells, immune cells, and endothelial/stromal cells. The final result of this process is a very tangled net of interactions promoting glioblastoma growth and progression. Nonetheless, recent data are suggesting that the microenvironment can also be a niche in which glioblastoma cells can differentiate into glial cells losing their tumoral phenotype. Here we summarize the known interactions between micro-environment and glioblastoma cells highlighting possible therapeutic implications.

## Introduction

The 2021 World Health Organization (WHO) Classification defines glioblastoma (GBM) as a diffuse astrocytic glioma without IDH (Isocitrate dehydrogenase) and H3R gene mutations, with enhanced microvascular proliferation, necrosis, and specific alterations like gain of chromosome 7/chromosome 10 loss, EGFR (Epidermal Growth Factor Receptor) amplification, and/or TERT (Telomerase Reverse Transcriptase) mutations (1, 2).

GBM is one of the most fatal primary central nervous system (CNS) malignancies with an estimated 5-years overall survival (OS) of only 6.8% (3, 4). In particular, the prognosis of patients with newly diagnosed GBM ranged from 12 to 18 months (5) while the recurrent disease is associated with a very poor outcome with an estimated OS of 5-10 months (6).

Several efforts have been spent to improve clinical outcomes of patients with GBM, nonetheless, none of the investigated agents have replaced the standard of care represented by maximal safe surgery followed by chemoradiation and adjuvant temozolomide, which has been adopted in 2005 (4). Even in the management of recurrent GBM, few systemic compounds demonstrated clinical efficacy (6).

There are several reasons behind these failures. First, the enrollment of GBM patients within clinical trials is limited only to10% of all GBM patients (7, 8).

However, the major cause explaining the difficult development of effective agents is certainly the complex biology of the disease. Indeed, GBM is associated with specific features which can be summarized in 1) the extremely high heterogeneity presented by tumor cells requiring combined treatment for different subtypes of GBM cancer cells; 2) the lack of biological models able to replicate or at least estimate the interaction between human tumor cells and the surrounding tissue; 3) the complex microenvironment surrounding the tumor (9–12). Furthermore, the presence of glioblastoma cancer stem cells (CSCs) has been employed to explain the impressive recurrence rate and regenerative proprieties of this tumor (13).

Of note, it has been demonstrated that GBM cells can manipulate the microenvironment surrounding themselves developing a niche sustaining tumor growth and development (14). This process involves immune cells, astrocytes, glial cells, neurons, extra-cellular matrix (ECM), vascular cells, and other cell types (15–20). There are several ways by which GBM can communicate with the surrounding tissue. The secretion of soluble factors able to modulate genomic expression and biological behaviors of tumor-associated cells is one of the most obvious and described systems.

Of interest, GBM cells can develop a nuclear and cytoplasmatic ‘‘continuum’’ with neighboring cells. These nanotubes mediate the transfer of protein and inorganic elements (21). Moreover, this also non-secretable molecules such as RNAs, DNA and also mitochondria, and nuclei can be transported from the tumoral to a surrounding cell by these nano-tubules (22–24). Notably, this system is also probably responsible for acquired resistance to radiation and systemic temozolomide (TMZ) (23).

GBM cells can also promote the creation of gap junction and cytoplasmatic connections (15–20). Finally, the secretion of microvesicles, extracellular vesicles, and exosomes can promote communication with also very distant cells or tissues (15–20).

The interactions between tumor cells and immune cells have acquired an increased interest due to the availability of agents able to promote immune-system reactivation. Nonetheless, other interactions have been only partially investigated and could hide novel promising targets for tumor treatment. Here we performed a review summarizing the identified interactions between GBM and its associated microenvironment. We also focused our attention on possible novel treatments targeting these complex interactions.

## Immune Cells and Glioblastoma

Immune-contexture assumes a very important role within the GBM microenvironment. It can stimulate the progression and development of tumor cells (25).

In contrast to what was supposed in the past, it has been largely demonstrated that glioblastoma is not an immune ‘‘cold tumor’’ (26, 27). Similar to other tissue, the CNS has its resident immune tissue represented mainly from microglia (28). Furthermore, like other solid tumors, GBM can activate and recall migrating immune cells from systemic tissues and lymphatic vessels (28). Indeed, there is a connection between deep cervical nodes, dural sinus, and lymphatic vessels allowing systemic immune cells to move into the CNS reaching the target site (29, 30). CNS is regulated by several molecular mechanisms able to enhance or suppress the immune response. However, the main difference with other peripheral non-CNS tissues is that the balance between inhibitory and stimulating mechanisms is biased in favor of immune suppression (31–35). One of the most important factors mediating an immune-inhibition is the TGFβ2 (transforming growth factor β2) (36, 37). This factor can mediate inhibition of Interleukin 2 mediated T cell survival and reduce the production of critical effector proteins by lymphocytes and other immune cells (36, 37). An increased intracranial pressure, such as that observed during an inflammation response, could be catastrophic and associated with irreparable damage to the neurological tissues. Thus, CNS protects itself preventing prolonged immune response with activation of several mechanisms supporting an immune-depressive status (31–35).

Recently, two transcription factors showed to mediate several immune-depressive effects in GBM (38). These factors are represented by SRY-Box Transcription Factor 2 (Sox2) and octamer-binding transcription factor 4 (Oct4) whose activation promotes the suppression of both innate and adaptive immune responses maintaining glioma cell stemness and tumor-propagating potential (38). In particular, the co-expression of Oct4/Sox2 inhibits the expression of CCL5 (C-C Motif Chemokine Ligand 5), CXCL9 (C-X-C Motif Chemokine Ligand 9), CXCL10, and CXCL11 which are essential to induce lymphocyte CD8 effector (Th1 response) attraction (38). Furthermore, they promote the secretion of SPP (signal peptide peptidase), IL8 (Interleukin 8), CXCL5, CCL20, IL6 (interleukin 6) inducing Treg (immune-inhibitory) response and shifting macrophage differentiation toward an immune-regulatory profile more than an immune-active one (38). Several immune checkpoints such as PD-L1 (Programmed Death Receptor Ligand 1), CD70 (Cluster of differentiation 70), A2aR (adenosine A2A receptor), and TDO (Tryptophan 2,3-dioxygenase) are also overexpressed in cells with Sox2/Oct4 overexpression (38). The effects promoted by Sox2/Oct4 are directly mediated by overexpression of the BRD3 (Bromodomain containing protein 3) and BRD4 (Bromodomain containing protein 4) proteins which belong to the Bromodomain and extra terminal motif (BET) proteins family. The BRD3 and BRD4 proteins act modulating the activity of the histone 3 (H3) acetylation mediated by the H3K27Ac enzyme. This mechanism appears of particular interest considering the availability of pan BET inhibitors.

Another recent research investigated RNA expression of GBM-derived sphere-line treated with the BET inhibitor JQ1. The inhibition of BET resulted in a significant modulation of genes responding to Interferon-alpha (enhanced in about 50% of GBM) through a direct transcriptional inhibition more than interference to the JAK (Janus Kinase) -STAT (Signal transducer and activator of transcription) pathway (39).

BET inhibitors have recently been assessed on phase I clinical studies and further trials assessing their clinical efficacy are needed (40–47).

In conclusion, differently from other tissues, CNS physiologically employs signals able to reduce an immune innate and adaptive response. This happened to prevent catastrophic effects associated with an uncontrolled inflammation in a dedicated system such as the brain or the spinal cord. In this context, GBM cells adopt several mechanisms to reinforce this inhibition. Glioblastoma stem cells (GSCs) are probably an important component of this complex mechanism as they express important transcription factors such as Sox2 and Oct4 (38). Nonetheless, it is also likely that other pathways converge on the same inhibition suggesting that the inhibition of a singular cascade could not be associated with an effective immune response reactivation.

### GBM – Microglia, Myeloid Cells, and Macrophages

Immune resident cells of the CNS are microglia and macrophages including perivascular, meningeal, choroid plexus, and circumventricular macrophages (28). These cells cover over 50% of the GBM tumor load and their composition change during the time and tumor progression (48). In the early phases of GBM development, the microglia is the most represented infiltrating cell subtype while macrophages and myeloid cells composed a large part of GBM volume in advanced phases (49, 50). Differently from macrophages, microglia constitute the resident immune system of the CNS. These cells can move within the CNS but they do not circulate in other tissues. Both microglia and macrophages are ineffective against tumors as their immune response is suppressed by the presence of an immune-depressive cytokines storm (11, 51). Indeed, GBM cells can directly mediate the production of several immune-depressive cytokines. Furthermore, cancer cells can manipulate the secretion and phenotype of surrounding immune cells shifting their phenotype toward an immune-suppressive one and initiating positive feedback leading to an immune-suppressive contexture. Notably, macrophages and myeloid cells are important protagonists of this process.

Myeloid cells constitute a large part of the immune-contexture of GBM. These cells are recruited directly from tumors cells and then can differentiate toward macrophages and monocyte phenotypes (52).

Myeloid cells have been classified into monocytic and granulocytic subtypes. Both these cells inhibit T cell and NK activities. Curiously, some data seem to confirm a negative prognostic role of these cells in males while these same cells can positively activate an immune response in female patients (52).

The main stimulation for macrophages and microglia accrual around the tumor is mediated by the same glioblastoma cells through the production of CCL2 (C-C Motif Chemokine Ligand 2), CCL7 (C-C Motif Chemokine Ligand 7), GDNF (Glial Cell-Derived Neurotrophic Factor), SDF1 (stromal cell-derived factor 1), TNF (tumor necrosis factor), VEGF (vascular endothelial growth factor), ATP (adenosine triphosphate), CSF-1 (Colony-stimulating factor 1), GM-CSF (Granulocyte-Macrophage Colony-Stimulating Factor), and expression of OLIG2 (Oligodendrocyte transcription factor 2) (48, 51, 53). Macrophages and microglia can perpetuate themselves accrual through the production of CCL2 resulting in a positive feedback loop (53). In advanced phases, microglia are localized mainly around tumor tissue while macrophages are localized in perivascular regions (54).

The differentiation of macrophages ranges from two specific phenotypes which are: M1 (immune-response enhancer) or M2 (immune-regulator inhibiting immune response) (55, 56). Although explicative, this classification is not definitive as macrophages can reach an intermediate grade of differentiation activating both genes of the M1 and M2 subtypes (57). In GBM, the resulting phenotype can inhibit immune response through secretion of transforming growth factor β1 (TGFβ1), arginase 1 (ARG1), or interleukin 10 (IL-10) enhancing neo-angiogenesis through VEGF production and extracellular matrix modeling by metalloproteases (MP) (38). On the other hand, these macrophages also produce pro-inflammatory molecules such as IL-β1 (interleukin β1), TNF, IL-5 (interleukin 5), and IL-12 (interleukin 12) which are molecules stimulating the immune response (38).

It has been demonstrated that macrophages promote GBM growth and progression in different ways (11).

First, the macrophage tumor suppression phenotype inhibits the response of other immune cells surrounding the tumor. This immune inhibition is mainly explicated by myeloid cells and differentiated macrophages which miss the activation of natural killer (NK) cells, the production of interferon γ (INF γ) and TGFβ (55). These same cells can induce lymphocytes differentiation toward an immune-regulatory (Th2) profile by TGFβ, reactive oxygen species (ROS), cysteine depletion, L-selectin downregulation, ARG1, and inducible nitric oxide synthase (iNOS2) production (36, 37).

The remodeling of ECM is essential for tumor migration and is mediated by the production of inactive metalloproteases enzymes by GBM cells (48, 58). These pro-Metalloproteases are then activated by enzymes produced by microglia. Moreover, the production of CSF1 by glioblastoma induces the release of the insulin-like growth factor-binding protein 1 (IGFBP1) by microglia which Is essential to promote vascularization and angiogenesis (48, 58, 59).

The administration of macrophages able to restore immune response would be a promising strategy able to partially restore immune-response against the tumor. The development of engineered macrophages (car-M) is at an early stage of assessment but it could be a promising strategy for GBM treatment (60).

Similar to macrophages and microglia also neutrophils and other innate immune cells are attracted by the tumor. In particular, inflammatory factors secreted after a surgical intervention such as IL-8, TNF, and CCL2 can promote neutrophil infiltration in addition to SDF1 and plasminogen activator inhibitor 1 secreted by tumor cells (61, 62). These innate immune cells can facilitate further accrual of macrophages and contribute to microenvironment remodeling.

### Lymphocytes and Antigen-Presenting Cells

Antigens exposure can start an adaptive immune response in the CNS like observed in other organs.

The presence of lymphocytes surrounding the tumor has been largely reported in GBM where their concentration correlates positively with survival (63, 64).

When an antigen is recognized by the immune system, a specific lymphocyte clone targeting the same antigen expands itself starting an adaptive immune response. The antigen-presenting cells (APCs) are essential to start the clonal expansion as these cells mediate the presentation of the antigen to the lymphocytes initiating the immune response (29, 30). Antigens captured within the CNS are processed by APC and then presented to lymphocytes probably in deep cervical nodes. Activated lymphocytes can move into the brain directly thanks to the increased permeability resulting from inflammation and neo-vessels or after exposure to antigen by APCs on the meningeal surface (29, 30).

Even if lymphocytes can move around GBM mass their ability to start an immune response against tumor is strongly inhibited by several factors. Once lymphocytes come to the peritumoral tissue they are invested by strong immune-depressive signaling mediated tumor cells and GBM associated microenvironment. The TGFβ1 and TGFβ2 molecules are the main characters for this inhibition (11, 37, 65).

It is important to observe that immune-inhibitory signals are largely provided by the microenvironment more than directly by GBM cells. This is a concrete example of how GBM cells manipulate the microenvironment to sustain their growth and expansion (11). Notably, it has been reported that GBM cells can induce pericytes to produce TGFβ and IL-10 (an inhibitory interleukin) (66).

GBM cells can also release directly inhibitory signals such Fas antigen ligand (FASLG), and other inhibitory molecules. Lymphocytes assume the classical exhaustion phenotype expressing several-inhibitory receptors including the PD-1, T cell membrane protein 3 (TIM3), lymphocyte activation gene 3 protein (LAG3), and T cell immunoreceptor with immunoglobulin and ITIM domains (TIGIT) (67).

As already specified, it is interesting to observe that a large part of inhibitory molecules released by GBM cells can be mediated by the Sox2/Oct4 (38).

The complex network of immune-inhibitory signals can partially explain the failure of clinical trials exploring immune-checkpoint inhibitors (ICIs). To date, three different phases III trials failed to show a clinical benefit from the addition of nivolumab in newly diagnosed and recurrent GBM (68–70).

In the last years, a novel immune strategy employing engineered T-cells (car-T) has been investigated with favorable preliminary results. In GBM the introduction of reprogrammed T-cells could be ineffective due to the immune shield provided by the tumor microenvironment (70). Strategies aimed to switch the composition of cancer-associated immune cells from an immune-suppressive to an immune-active one are of critical importance. Combinatory strategies employing more immune targets are under investigation (70).

## Neurons and Glioblastoma

The brain is composed of over 60% of the white matter which is largely composed of myelinic axons. Neurons, astrocytes, and oligodendrocytes with other glial cells (including microglia) are the most represented cells in the brain and CNS (71).

The study of the interactions between these components and glioblastoma appears of extreme interest as these are strictly related to some of the most important biological and clinical features of GBM cells.

Indeed, connexons between white matter, neurons, and tumors can partially explain the high recurrence rate of the disease as well as the ability to relapse also in a distant site such as the contralateral hemisphere (71–74). Of interest, the interaction between neurons and GBCs assumes a particular interest in recent years.

Indeed, the biological niche composed of GBM cells, GSCs, peritumoral oligodendrocytes, astrocytes, and also neuronal axons can both drive differentiation of a GSCs cell toward a tumoral phenotype or, surprisingly, toward a differentiated non-tumoral cell subtype (75). On the other hand, also GBM cells can induce the dedifferentiation of astrocytic cells while astrocytes and oligodendrocytes can support tumor growth in different ways. In particular, astrocytes can support tumor growth and development through the secretion of several cytokines and other soluble factors stimulating directly GBM growth and maintaining an immune-suppressive contexture (76, 77).

### Astrocytes

Tumor-associated astrocytes (TAAs) can contribute to GBM growth in different ways. During early phases of tumor development, TAAs respond to initial injury with the secretion of TGFβ, IL-6, and Insulin growth factor 1 (IGF-1) which could contribute to GBM sustainment (76, 77). These same TAAs express the sonic hedgehog (SHH) gene and are concentrated in the perivascular niche of GBM (78–80). In early and advanced phases communications between GBM and TAAs occurred especially through the secretion of extracellular vesicles which contribute to stimulating several growth factors by TAAs (81). These include the vascular endothelial growth factor (VEGF), fibroblast growth factor (FGF), hepatocyte growth factor (HGF), and epidermal growth factor (EGF) (81).

The contribution of TAAs is of key importance as these cells can stimulate neo-angiogenesis through secretion of VEGF and hypoxia-inducible factor -1 (HIF-1) (82, 83).

Of interest, it has been demonstrated that astrocytes actively participate in chemotherapy resistance by GBM (84). The exact mechanism by which this is possible is still unclear but *in vitro* studies identified that chemoprotection performed by TAAs is possible only when these cells are connected to cancer cells by gap junctions. Indeed, apoptosis and GBM cells death increased when the development of gap junctions was inhibited by the administration of carbenoxolone (a gap junctions communication inhibitor) (84).

One of the most surprising behaviors of astrocytes is the capacity to be transformed in tumor-initiating cells. *In vivo* studies demonstrated that several oncogenes including MYC, RAS, and EGFR variant III can dedifferentiate astrocytes in tumoral cells (85, 86). This close relationship suggests a common phenotype shared by GBM and astrocyte supporting the hypothesis that astrocytes are the precursor normal cells by which gliomas originate. It is still unknown the exact mechanism, but it has been observed that glioma cells inhibit the expression of the onco-suppressor p53 in normal astrocytes (87, 88). The malignant transformation of astrocytes required the miR-10b which is silenced in TAAs but is largely expressed and secreted by GBM cells using extracellular vesicles (89).

Although the already cited oncogene (MYC, RAS, and EGFR) can mediate the transformation of astrocyte into malignant glioma cells it is still unclear the exact mechanism by which GBM cells can initiate this process in humans (85, 86). However, the manipulation and conversion of surrounding astrocytes into neoplastic cells can be another issue explaining the well-known recurrence and regeneration proprieties of this tumor

### Neurons

Neurons can be manipulated by glioma cells to stimulate tumor progression and invasion. One of the first documented interactions between neurons and GBM is associated with the release of glutamate which could be enhanced in patients with GBM resulting in seizure onset and increased vascular permeability mediated by the N-methyl-D-aspartate receptors (NMDA) (90, 91). Of interest, synapsing signaling of cortical neurons can directly stimulate GBM growth. Neurons and oligodendrocyte precursor cells can secrete the postsynaptic adhesion molecule neuroligin 3 which mediated the activation of several key pathways including the mammalian target of rapamycin (mTOR), phosphoinositide-3-kinase (PI3K), and focal adhesion kinase (FAK) (92). Also, the brain-derived neurotrophic factor chaperone Bip (HSPA5) has been identified as an agent able to stimulate mitosis and cell division (92).

### Oligodendrocytes and White Matter

Oligodendrocytes may absolve an inhibitory function against GBM cells. Indeed, these cells can activate the WNT inhibitory factor 1 leading to the inhibition of GBM growth and proliferation (93, 94). In CNS, Oligodendrocytes modulate neural plasticity, metabolic support, and axon activity through myelination. It is well known that GBM can spread through myelinic axons (75). The butterfly shape commonly observed in GBM indicates that tumor cells can invade the contralateral side following the commissure fibers of the corpus callosum (75). Similarly, GBM can spread through the arcuate fasciculus and using the radiation of the corpus callosum (75). It has been supposed that GBM cells adopted myelinated axons as scaffolds and tracks however a recently published study could drastically modify this concept (75). Glioblastoma stem cells are contained in a specific biological niche composed of their microenvironment which influences GSCs fate and differentiation. It is easy to think that GSCs differentiate into cancer cells but it is not always true as in some conditions these cells can differentiate into normal cells without tumorigenicity. It seems that a biological niche composed of white matter can induce GSCs to differentiate toward a pre-oligodendrocyte phenotype losing oncogenic potential (75). This mechanism is similar to that happening during the response to injury (75). Thus, this is an important discovery demonstrating that GSCs can differentiate toward a normal cell phenotype.

The mechanism by which this happens involves the white matter destruction which leads to upregulation of the SOX10 transcription factor. The SOX10 mediates oligodendrocyte differentiation also by cancer cells (75).

Further investigation of this pathway will probably provide new therapeutic insights for the clinical management of GBM.

## Cancer Stem Cells

Glioblastoma stem cells explain the extreme clinical aggressiveness of GBM. In GSCs are associated with the high recurrence rate of the tumor even after complete resection (72). These cells are also associated with tumor renewal and resistance to treatment (73). Finally, the ability to spread and relapse in a distant site of the brain such as the contralateral hemisphere could be partially explained by GSCs an interaction between tumors and neurons (71–74). As already discussed, the differentiation of GSCs could be shifted toward a tumoral or non-tumoral phenotype according to the biological niche in which these cells are located (75).

Glioblastoma stem cells are localized in a specific niche localized in the perivascular and hypoxic regions of the tumor. These cells are similar to normal neural stem cells (NSCs) which are localized mainly in the subventricular zone of the brain which is a common site of origin for glioblastoma (95–97).

It is still unclear if GSCs derived from altered NSCs or mutated glioma cells (98). Glioblastoma Stem Cells are surely associated with GBM progression and recurrence after surgery (99, 100). The surface marker CD133 is one of the most adopted markers to recognize GSCs.

The expression of CD133 is regulated by Sox2 and agents targeting or interfering with this transcription factor can reduce tumor-initiating ability, resistance to chemotherapy, and recurrence (101). Nestin is another upregulated factor reported on GSCs that is directly correlated with poorer survival (98) (102).

Notably, the specific position of GSCs leads to an interaction with Endothelial cells (ECs) and pericytes. It has been supposed that GSCs directly respond to hypoxia stimulating vessels creation and neo-angiogenesis through the production of VEGF (103, 104). Moreover, GSCs can differentiate into ECs and pericytes (103–105). The most surprising finding related to GSCs is that these cells can be located at a distance of up to 3 cm from the primary tumor (106). Moreover, their histological identification is almost impossible as these cells are indistinguishable from normal tissue cells (72). Nonetheless, some studies suggest that GSCs derived from the GBM core are different from those isolated by peritumoral tissue presenting different behaviors in terms of proliferative potential and expression of stem-cell markers (72, 107, 108). Even if peritumoral GSCs are less aggressive compared to GSCs from the core of GBM these same cells are also more resistant to temozolomide and radiation therapy (72, 107, 108). As discussed in a previous paragraph, the transcription factors Sox2 and Oct4 are activated in GSCs promoting glioma cell stemness and stimulating several mechanisms leading to innate and adaptive immune response inhibition (38). Curiously, recent data suggest a strong interaction between glioblastoma cancer cells and GSCs (109). This is mainly mediated by the cascade activated by the brain-derived neurotrophic factor (BDNF) secreted by GBM cells and the receptor neurotrophic receptor tyrosine kinase 2 (NTRK2) localized on stem cells. This interaction leads to a paracrine effect resulting in tumor growth and development (109).

## Endothelial Cells

Neovascularization and new vessels development are both hallmarks of GBM. The fast growth of the tumor mass required high blood intake thus there are several identified pathways by which GBM cells can interact and manipulate ECs activity (110).

Hypoxia is one of the most important stimulations for tumor growth, vessels development, and acquisition of more aggressive pathological features by tumor cells (111–113). In general, hypoxia leads to a metabolic switch by tumor cells which are more likely to promote aerobic glycolysis. Hypoxia induces also an attenuated expression of DNA repair enzymes and impedes the formation of Reactive Oxygen Species (ROS) reducing the cytotoxic effect of radiation therapy (111–113).

Notably, ROS can assume a different biological role according to their concentration. Low levels of ROS are associated with stimulation of hormone secretion, synaptic plasticity, and immune response (114, 115). High levels of ROS are instead associated with DNA damage and in particular p53 damage (116). The DNA damage promoted by ROS could partially explain the switch from low to high-grade gliomas (117). The NADPH oxidase (NOX) 4 is activated by PDGF and TGF-β and is a key enzyme associated with ROS production (H2O2) (118). The inhibition of this enzyme could be a promising target in patients with GBM.

One of the most established pathways for new vessels development is the recruitment of EC progenitors by the bone marrow. This is a process occurring during embryogenesis or after an ischemic insult which is also adopted by GBM cells (119, 120). Tumor cells or microenvironments manipulated by GBM cells can secrete the SDF-1 which is the ligand of the CXCR4 receptor expressed by EC progenitors (121, 122). The interaction between SDF-1/CXCR-4 resulted in EC activation and recruitment of novel EC (121, 122).

It has been well established that GBM cells and the surrounding microenvironment can stimulate the production of the VEGF. This factor can drive EC toward the development of novel vessels in a process known as sprouting angiogenesis. Notably, the neoangiogenic promotion carried out by VEGF can be inhibited by the interaction between the Notch receptor and Delta-like canonical Notch ligand 4 (DLL4) (123). Curiously, novel vessels development can originate also through a process known as vasculogenic mimicry (124–126). This phenomenon is mediated by the same tumor cells which can differentiate to create a vessels-like structure. In particular, macrophages surrounding tumors can mediate cyclooxygenase-2 (COX-2) activation stimulating vasculogenic mimicry (127). Other supposed mechanisms involved in this pathway are the expression of VE-Cadherin by GBM stem cells resulting from hypoxia and the HIF 1 and HIF 2 secretion. Also, the mTOR expression seems to be involved in this pathway (128).

Vascular co-option is another mechanism by which tumor cells move around pre-existing vessels gaining the access to oxygen and nutrients. Curiously GBM cells can induce secretion of bradykinin by EC cells. Bradykinin plays a chemotaxis effect on GBM cells (129, 130). The interaction between SDF1/CXCR4 (129, 130) and the expression of the EGFRvIII are also involved in this mechanism (129–131).

A well-described mechanism associated with neo-angiogenesis is the interaction between VEGF and VEGFR2 and VEGFR1 (132). The interaction between VEGF and VEGFR1 or VEGFR2 induces the phosphorylation and activation of the ERK 1/2 (extracellular signal-regulated kinases 1 and 2) and p38 MAPK (mitogen-activated protein kinase) which together mediated transcription of pro-angiogenic factors (132).

This interaction also results in blood vessels permeability, and proteins lost from blood (110) explaining an increased permeability, edema onset, and increased intracranial pressure. This can explain the clinical benefit experienced by patients treated with the anti-VEGF agent bevacizumab (133–136). Even if the administration of bevacizumab is associated with reduced edema and vascularization within tumor masses it failed to show a significant survival benefit among patients with GBM (133–136).

The lack of the survival benefit observed could reflect the co-existence of several mechanisms resulting in angiogenesis and vessels development. In this optic, the inhibition of the VEGF mediated by bevacizumab can activate or reinforce other molecular pathways converging on angiogenesis promotion resulting in resistance to the anti-VEGF.

The study of the interactions between immune cells in tumor-associated microenvironment assumes an increased interest (137). Increasing data seem to indicate that hypoxia induces secretion of the VEGF which is one of the main molecules mediating this signaling. In particular Regulatory T lymphocytes (T reg) mediate the production of IL-10, IL-4, and IL-13 inducing differentiation of macrophages into M2 phenotype and stimulating expression of inhibitory B7-H receptor on their membranes (137).

Thus, the inhibition of VEGF could also result in an enhanced immune response against the tumor. This supposed synergic effect has led to the assessment of combination strategies in the clinical setting. Unfortunately, a phase II study assessing the combination between bevacizumab and pembrolizumab (a PD-1 inhibitor) failed to show a significant clinical efficacy on patients with GBM (138).

## Targeting the Microenvironment in Glioblastoma

There are very few systemic agents showing clinical efficacy in patients with glioblastoma (139–142). As already discussed in previous paragraphs, a great interest in the immune contexture of GBM is explained by the availability of active compounds able to restore immune response against tumors. Nonetheless, immune-checkpoint inhibitors failed to improve the survival of patients with GBM (68). The reason for this failure can be partially explained by the lack of uniformly expressed tumor-specific antigen due to the high heterogeneity of tumor GBM cells as well as the presence of an immune-depressive microenvironment which impairs the ability of immunotherapy to work. Also, GSCs can mediate immune escape (38).

Novel combination strategies are under investigation to overcome these limitations.

For example, it has been demonstrated that other immune checkpoints co-exist with PD-1. These are the indoleamine 2,3-dioxygenase (IDO1), T cell immunoglobulin-mucin-domain containing-3 (TIM-3), and lymphocyte activation gene 3 (LAG3) (143). Novel trials exploring combinations between PD-1 and LAG3 (NCT02658981) or IDO1 (NCT03707457) are under investigation. Similarly, the combination between temozolomide and IDO1 inhibitors is under investigation (NCT02052648).

Previous trials investigating neoantigen vaccination showed that vaccines can induce a significant increase of tumors infiltrating lymphocytes (144). Unfortunately, these infiltrating lymphocytes assume an exhausted phenotype. Thus the co-administration of multi-epitope vaccines and immune-checkpoint inhibitors could be another promising approach (NCT02149225 GAPVAC trial) (144). Strategies assessing co-administration of vaccines and CAR-T engineered with EGFRvIII and PD-1 are also under investigation (NCT04003649, NCT04201873, NCT02529072, NCT02287428) (145–147). Also, the administration of co-stimulatory agonists able to enhance T cell function is under investigation. Agonists such as CD27, 4-1BB, OX40 or CD40 are under investigation (NCT04547777, NCT02658981, NCT03688178, NCT04440943) (145–147).

Since macrophages are strongly associated with the development of an immune-depressive microenvironment the possibility to develop an engineered cell type of macrophage could be a promising strategy allowing a microenvironment to switch toward an immune-active phenotype (60). This Car-M strategy is still under early assessment however this appears a very promising approach.

As already specified SOX2 and Oct4 are two transcription factors able to modulate a very large amount of genes involved in the immune response. These two factors act through proteins belonging to the BET family. To date, BET inhibitors have been assessed on phase I trials with more of them showing a safety profile (39–47). A further investigation of these agents could be important for patients with GBM.

The inhibition of the CSF1 receptor can inhibit the interaction between GBM and cells of the immune innate system including tumor-associated macrophages. Studies on murine models showed that macrophages develop resistance to CSF1 inhibition through the expression of the insulin growth factor 1 (148). The co-administration of CSF1 and IGF1 inhibitors could be an interesting approach as associated to restored immune-active microenvironment resulting from macrophages activation (148).

In the last years, several agents targeting angiogenesis have been tested in GBM without significant results. The only FDA-approved drug is bevacizumab which has been associated with prolonged progression-free survival, a reduction of symptoms related to the tumor but failed to show a significant impact on overall survival (133–136). Several efforts are spent to understand the reason for bevacizumab resistance. The very high hypoxia level described inside the tumor can partially explain this failure. Indeed, hypoxia is associated with upregulation of the hypoxia-inducible protein 2 (HIG2) gene with downregulation of the CYLD gene expression. The overexpression of HIG2 resulted in HIF-1β, VEGF expression, and bevacizumab resistance through direct stimulation of hypoxia-inducible factor. Nonetheless, agents targeting HIG2 products failed to improve the clinical outcomes of patients with GBM (149). Novel approaches are aimed to target the angiotensin II receptors (AngII-R) and VEGF (150). However, this approach has been evaluated only in murine models thus assessments on humans are necessary to further assess this approach.

Since GBM cells adopt extracellular vesicles to communicate with surrounding tissue, a system able to reduce vesicles uptake and synthesis can be a promising target. Pre-clinical studies showed that inhibitors of neutral sphingomyelinase (GW4869) can reduce the production of extracellular vesicles while heparin and annexin A1 inhibitors can reduce vesicles intake by target cells (151–153).

Direct inhibition of junctions between GBM and microenvironment is also a strategy under investigation. Inhibition of connexin 43 is essential to inhibit the interaction between GBM and astrocytes (154). Another interaction between oligodendrocytes, astrocytes, and GBM cells is the interaction between neuroligin 3/ADAM10 (155). Indeed, GBM cells produce neuroligin 3 which is cleaved from the ADAM10 secreted by neurons and oligodendrocytes (156). The effect is a stimulation of GBM growth and development (11). Thus inhibitors of the ADAM10 appear a promising strategy for GBM which should be further assessed among clinical trials in humans.

## Conclusion

GBM remains a fatal disease with limited treatments. The reason for this failure could be partially explained by the development of a complex and effective net of interactions between tumor cells and surrounding tissue cells. The microenvironment resulting from the manipulation carried out by cancer cells can feed and stimulate GBM proliferation hiding and protecting tumor cells from systemic treatments.

Interactions between tumor, endothelial and immune cells are under careful assessment due to the availability of drugs targeting these pathways. Recently, also the associations between glioblastoma and astrocytes, oligodendrocytes, white matter, and neurons come out from the shadow offering novel promising targets with therapeutic implications.

Due to the presence of deep communications between tumor cells and the microenvironment the use of agents targeting more than one altered intracellular cascade at the same time appears a promising approach.

In this optic novel immune-combination including immune checkpoint inhibitors) targeting PD-1/PD-L1, IDO1, TIM3, LAG3), engineered immune cells (CAR-T and CAR M), immune agonists (targeting OX40, CD27,4-1BB, CD40), and BET inhibitors are under investigation. Notably, tumor-associated macrophages are one of the most important cell-associated with the development of an immune-depressive habitat. Thus, a strategy able to reverse this effect such as CAR M assumes a particular interest.

Strategies aimed to inhibit the signaling between GBM cells and the microenvironment are also of key importance as able to inhibit the manipulation carried out by tumor cells on surrounding tissues. Inhibitors of sphingomyelinase, annexin A1 can act on extracellular vesicles secretion and intake while other agents such as ADAM10 inhibitors could directly interfere with the jap junctions connecting GBM to other cells.

Finally, it should be noted that a specific microenvironment composition (such as that described on the white matter) can promote GBM differentiation and convert a tumor cell into an oligodendrocyte well-differentiated element. This surprising finding suggests that there are some elements inside the microenvironment that can provide inhibitory messages to GBM reversing its natural course. Interactions between neurons and glia (especially oligodendrocyte) appear of particular interest and should be further assessed as could hide important targets for novel drugs development. In conclusion, GBM should be considered as a network of interactions in which an action perpetuates against tumor cells result in a response of the associated microenvironment and vice versa. The role of the microenvironment should be always considered during pre-clinical studies and would offer novel targets for patients with GBM.

## Author Contributions

VN and LG: writing and draft. EF, AB, AT, and SB: project conception and reviewing. All authors contributed to the article and approved the submitted version.

## Conflict of Interest

The authors declare that the research was conducted in the absence of any commercial or financial relationships that could be construed as a potential conflict of interest.

## Publisher’s Note

All claims expressed in this article are solely those of the authors and do not necessarily represent those of their affiliated organizations, or those of the publisher, the editors and the reviewers. Any product that may be evaluated in this article, or claim that may be made by its manufacturer, is not guaranteed or endorsed by the publisher.

## Glossary

ADAM10: Adam metallopeptidase domain 10
APC: Antigen Presenting Cell
ARG1: arginase 1
A2aR: adenosine A2A receptor
ATP: adenosine triphosphate
BDNF: brain derived neurotrophic factor
BET: Bromodomain and extra terminal motif proteins family
BRD3: Bromodomain containing protein 3
BRD4: Bromodomain containing protein 4
CD70: Cluster of differentiation 70
CD133: prominin-1
CCL2: C-C Motif Chemokine Ligand 2
CCL5: C-C Motif Chemokine Ligand 5
CCL7: C-C Motif Chemokine Ligand 7
COX 2: cyclooxygenase-2
CCL20: C-C Motif Chemokine Ligand 20
CXCL3: C-X-C Motif Chemokine Ligand 3
CXCL5: C-X-C Motif Chemokine Ligand 5
CXCL9: C-X-C Motif Chemokine Ligand 9
CXCL10: C-X-C Motif Chemokine Ligand 10
CXCL11: C-X-C Motif Chemokine Ligand 11
CNS: Central Nervous System
CSF-1: Colony stimulating factor 1
DLL4: Delta-like canonical Notch ligand 4
EC: Endothelial cell
ECM: Extra-cellular matrix
EGF: Epiderma growth factor
EGFR: Epidermal growth factor receptor
ERK 1/2: extracellular signal-regulated kinases 1 and 2
FAK: focal adhesion kinase
FGF: Fibroblast Growth factor
GBM: Glioblastoma
GDNF: Glial Cell Derived Neutrophic Factor
GM-CSF: Granulocyte-Macrophage Colony-Stimulating Factor
GSC: Glioblastoma stem cell
JAK: Jasus Kinase
HGF: Hepatocyte growth factor
HIF-1: hypoxia inducible factor 1
HIF-2: hypoxia inducible factor 2
HIG-2: hypoxia inducible protein 2
HSPA5: brain-derived neurotrophic factor chaperone Bip
IDH: Isocitrate dehydrogenase
IDO1: Indoleamine 2,3-dioxygenase
IGF1: insulin growth factor 1
IGFBP1: insulin-like growth factor-binding protein 1
IL5: Interleukin 5
IL6: Interleukin 6
IL8: Interleukin 8
IL10: Interleukin 10
INF γ: interferon γ
iNOS: inducible nitric oxide synthase
LAG 3: lymphocyte activation gene 3
MAPK: mitogen-activated protein kinase
MGMT: O^6^ – methylguanine DNA methyltransferase
MP: Metalloprotease
mTOR: mammalian target of rapamycin
NMDA: N-methyl-D-aspartate receptors
NOX 4: NADPH oxidase 4
NSC: Neural stem cell
NTRK2: neurotrophic receptor tyrosine kinase 2
Oct4: Octamer-binding transcription factor 4
OLIG2: Oligodendrocyte transcription factor 2
OS: Overall survival
PD-1: Programmed Death Receptor 1
PD-L1: Programmed Death Receptor Ligand 1
PI3K: phosphoinositide-3-kinase
ROS: reactive oxygen species
SDF1: stromal cell-derived factor 1
SHH: Sonic Hedgehog
SOX2: SRY-Box Transcription Factor 2
SOX10: SRY-Box Transcription Factor 10
SPP: Signal Peptide Peptidase
STAT: Signal transducer and activator of transcription
TAA: Tumor associated astrocytes
TDO: Tryptophan 2,3-dioxygenase
TERT: Telomerase Reverse Transcriptase
TGFβ1: transforming growth factor β1
TIM-3: T cell immunoglobulin-mucin-domain containing3
TMZ: Temozolomide
TNF: tumor necrosis factor
VEGF: vascular endothelial growth factor
WHO: World Health Organization
